# Influence of metastatic site as an additional predictor for response and outcome in advanced colorectal carcinoma

**DOI:** 10.1038/sj.bjc.6990287

**Published:** 1999-04

**Authors:** L Assersohn, A Norman, D Cunningham, T Benepal, P J Ross, J Oates

**Affiliations:** Gastrointestinal Unit, The Department of Medicine, Royal Marsden Hospital, Downs Road, Sutton, UK

**Keywords:** colorectal cancer, metastatic site, prognostic, chemotherapy

## Abstract

Every year, 31 230 men and women are diagnosed with colorectal carcinoma, and up to 60% of these will ultimately develop advanced disease. However, there is little information to identify which patients are most likely to benefit from palliative chemotherapy. This analysis is unique in evaluating how the site of metastasis influences response and survival. A database of 497 patients treated within randomized clinical trials using 5-Fluorouracil (5FU)-based chemotherapy at the Royal Marsden Hospital was analysed. The potential for site of metastasis as a predictive variable for response to chemotherapy and survival was examined, in addition to other clinical parameters. The presence of liver metastases was a better predictor for overall response than either performance status or number of metastatic sites on presentation. Probability of response was significantly decreased by a raised serum carcinoembryonic antigen (CEA) and presence of peritoneal metastases. In liver metastases, a normal serum albumin was as significant a predictor for response as good performance status. The most important predictor for survival was initial performance status. The number of metastatic sites on presentation had no influence on survival. Site of metastasis can predict for response to 5FU-based chemotherapy and patients should be stratified according to the involved site of metastasis in the future. © 1999 Cancer Research Campaign


*British Journal of Cancer* (1999) **79**(11/12), 1800–1805
© 1999 Cancer Research Campaign
Article no. bjoc.1998.0287

### Influence of metastatic site as an additional predictor for response and outcome in advanced colorectal carcinoma

**L Assersohn, A Norman, D Cunningham, T Benepal, PJ Ross and J Oates**

Gastrointestinal Unit, The Department of Medicine, Royal Marsden Hospital, Downs Road, Sutton, UK

**Summary** Every year, 31 230 men and women are diagnosed with colorectal carcinoma, and up to 60% of these will ultimately develop advanced disease. However, there is little information to identify which patients are most likely to benefit from palliative chemotherapy. This analysis is unique in evaluating how the site of metastasis influences response and survival. A database of 497 patients treated within randomized clinical trials using 5-Fluorouracil (5FU)-based chemotherapy at the Royal Marsden Hospital was analysed. The potential for site of metastasis as a predictive variable for response to chemotherapy and survival was examined, in addition to other clinical parameters. The presence of liver metastases was a better predictor for overall response than either performance status or number of metastatic sites on presentation. Probability of response was significantly decreased by a raised serum carcinoembryonic antigen (CEA) and presence of peritoneal metastases. In liver metastases, a normal serum albumin was as significant a predictor for response as good performance status. The most important predictor for survival was initial performance status. The number of metastatic sites on presentation had no influence on survival. Site of metastasis can predict for response to 5FU-based chemotherapy and patients should be stratified according to the involved site of metastasis in the future.

**Key words:** colorectal cancer; metastatic site; prognostic; chemotherapy

Colorectal carcinoma is the second most common malignancy in the UK, with 31 230 men and women being diagnosed in 1995 (Cancer Research Campaign, 1998). At least 40% of these patients ultimately develop metastatic disease (Mayer, 1992) and a further 20% develop locally advanced disease. Palliative cytotoxic chemotherapy has been shown to prolong survival and improve quality of life in several randomized studies (Nordic Gastrointestinal Tumour Adjuvant Therapy Group, 1992; Scheithauer et al, 1993; Allen-Mersh et al, 1994) but new treatment regimens are being sought to increase efficacy as the response rate is relatively poor (Poon et al, 1989; Advanced Colorectal Cancer Meta-Analysis Project, 1992).

With the development of more intensive treatment regimens, a better understanding of the clinical parameters that can predict response to chemotherapy is required. However, there is little information on predicting which patients would benefit most from treatment. In addition, by failing to stratify patients, some clinical trials inappropriately promote or dismiss new treatment regimens. The identification of factors predicting for response would enable more accurate comparisons to be made between different therapeutic approaches.

To date, there has been little investigation into the parameters that predict for response to chemotherapy, although it has been recognized that poor performance status predicts for lack of response to treatment (Steinberg et al, 1992). However, some authors have studied those clinical parameters that might predict for decreased survival, rather than response, such as poor differentiation of primary tumour (Finan et al, 1985; Stangl et al, 1994; Webb et al, 1995), low albumin (Finan et al, 1985; Steinberg et al, 1992; Fountzilas et al, 1996) and raised alkaline phosphatase (Chang et al, 1989). Some studies have demonstrated that a raised serum carcinoembryonic antigen (CEA) on presentation carries a poor prognosis (Chang et al, 1989; Webb et al, 1995), while others found there to be no significant correlation (Kouri et al, 1992, 1993).

So far, no studies have been performed specifically to evaluate the importance of metastatic site as a prognostic indicator for response to treatment and survival. There have been reports within some studies that hepatic deposits are the most likely to respond and peritoneal the least (Kouri et al, 1993; Hill et al, 1995*a*) but no further evaluation of this potentially important prognostic indicator has been performed. To determine whether metastatic site, along with other clinical parameters, influences response rate and survival in advanced colorectal carcinoma, we performed an analysis of a cohort of 497 patients who had been entered into prospective, randomized clinical trials (Hill et al, 1995*a*, 1995*b*; Seymour et al, 1996; Ross et al, 1997).

### METHODS

### Patient eligibility

This study used a prospective database that had collected data in the period from February 1990 to June 1996. The patients had been in randomized clinical trials (Hill et al, 1995*a*, 1995*b*; Seymour et al, 1996; Ross et al, 1997) with similar entry criteria

*Received 6 July 1998*
*Revised 19 September 1998*
*Accepted 14 October 1998*

*Correspondence to:* D Cunningham and well-balanced pretreatment characteristics in the different treatment arms. All patients had either inoperable locally-advanced or metastatic histologically-proven colorectal adenocarcinoma. Some patients included in the group with peritoneal disease also had omental disease. They were required to have adequate bone marrow (platelets > $100 \times 10^9$ $l^{-1}$ white cell count > $3 \times 10^9$ $l^{-1}$), renal (creatinine < 132 mmol $l^{-1}$, urea < 10.7 mmol $l^{-1}$) and hepatic (bilirubin < 30 mmol $l^{-1}$) function, life expectancy of at least 3 months and no previous malignancy or concurrent uncontrolled medical illness. All were intention-to-treat trials and, therefore, although performance status was 0–2 at the time of randomization, the performance status could have deteriorated to 4 by the time of first course of treatment.

### Pretreatment evaluation

Staging was performed by clinical assessment, full blood count (FBC), renal and liver function tests, CEA and computed tomography (CT) of the thorax, abdomen and pelvis. In addition, the diagnostic histological sample was reviewed by a histopathologist at the Royal Marsden Hospital. Performance status was assessed according to World Health Organization (WHO) guidelines (Miller et al, 1981).

### Chemotherapy

In all patients this was the first palliative treatment. Those who had received prior adjuvant chemotherapy were eligible only if this had been completed more than 12 months previously. 5-Fluorouracil (5FU)-based chemotherapy was given to all patients and the regimens used were:

(i) protracted venous infusion 5FU (300 mg $m^{-2}$) with or without interferon alpha (IFN) ($n$ = 158) (Hill et al, 1995*a*)
(ii) protracted venous infusion 5FU (300 mg $m^{-2}$) with or without mitomycin C ($n$ = 195) (Ross et al, 1997)
(iii) 5-day infusional 5FU (750 mg $m^{-2}$ $day^{-1}$) followed by weekly bolus 5FU (750 mg $m^{-2}$) with or without IFN ($n$ = 110) (Hill et al, 1995*b*)
(iv) leucovorin (200 mg $m^{-2}$) over 2 h then 5FU (400 mg $m^{-2}$) bolus and 5FU (400 mg $m^{-2}$) by infusion over 22 h on day 2, fortnightly with or without IFN ($n$ = 34) (Seymour et al, 1996).

### Evaluation of response

Tumour site, size and response was evaluated using CT scans at 10–12 weeks, and then after a further 10–12 weeks if there had been a response. Tumour response was assessed according to the WHO guidelines (Miller et al, 1981).

### Statistical methods

Different potential prognostic subgroups of patients were defined as a result of clinical experience and previous literature: age, sex, performance status, site of primary tumour, differentiation of primary tumour, Dukes staging at diagnosis, mode of administration of 5FU, site of metastasis, number of metastatic sites and serum CEA, alkaline phosphatase and albumin on presentation. As complete data on all clinical parameters were not available for every patient, the sample size varied, with a minimum sample size of 420 patients, unless individual sites of metastases were examined. When disease was not evaluable at a particular site, the patient was excluded from that site analysis.

Univariate analyses were performed using the chi-squared ($\chi^2$) test to assess the parameters which had individual statistical significance on overall response rate and response for different sites. Multivariate analysis was performed using step-wise logistic regression to evaluate the potential prognostic factors and their independent influence on overall response rate and response rate at different metastatic sites. As performance status and the mode of administration of 5-Fluorouracil are known to be strong predictors for response, these factors were forced to remain in the multivariate analysis model for response at individual sites of metastatic disease, effectively stratifying for these factors. Hazard ratios for overall survival according to different variables was calculated using Cox proportional hazards model. Results were considered significant if the $P$ value was less than 0.05.

### RESULTS

The data for 497 patients who were treated within prospective randomized studies between February 1990 and June 1996 was analysed in December 1997. Patient characteristics are shown in Table 1. There were 313 men and 184 women with a median age of 61 years (range 16–82 years). Sixty-six patients were alive at the last follow-up with a median follow-up of 789.5 days (range 57–2305 days). Median survival was 333 days with 45.9% of patients alive at 1 year, 16.7% alive at 2 years and 9.2% alive at 3 years.

Table 2 demonstrates the most favourable response at each metastatic site. 40% of patients with metastatic disease in the liver had a complete response (CR) or partial response (PR) while a CR or PR was achieved in 20.8% of lung metastases, 18.7% of nodal disease and 15.3% of locoregional disease. Only 10.4% of patients with peritoneal disease achieved a PR, with 68.7% having stable disease (SD) and 20.9% having progressive disease (PD). Disease was the most difficult to assess in patients with peritoneal metastases (26.4%).

### Univariate analysis

Univariate analysis was performed to assess the statistical significance of various factors on response to treatment (Table 3). Eight patients were not evaluable for overall response and were, therefore, excluded from the analysis. The significant positive prognostic indicators for response were, in decreasing order, the presence of one metastasis or locally advanced disease, albumin ≥ 35 g $l^{-1}$, performance status of 0 or 1, administration of 5FU by protracted venous infusion (PVI) and alkaline phosphatase < 133 U $l^{-1}$. Peritoneal ($P$ = 0.006) and lung ($P$ = 0.015) metastases were significantly less likely than other sites of disease to respond to chemotherapy. Age, sex, site of primary, differentiation of primary, Dukes stage (not shown) and initial CEA did not predict for response to chemotherapy.

### Multivariate analysis

#### *Overall response*

A total of 420 patients were evaluable for overall response (Table 4). The presence of liver metastases was the most important factor

© Cancer Research Campaign 1999

**Table 1** Patient characteristics

| Patient characteristics | *n* |
|---|---|
| Sex | |
| Male | 313 |
| Female | 184 |
| Performance Status | |
| 0 | 113 |
| 1 | 244 |
| 2 | 111 |
| 3 | 21 |
| 4 | 1 |
| Missing value | 7 |
| Site of Primary | |
| Colon | 324 |
| Rectosigmoid/rectum | 172 |
| Missing value | 1 |
| Differentiation | |
| Well | 21 |
| Moderate | 388 |
| Poor | 64 |
| Unknown | 24 |
| Dukes Stage | |
| A | 9 |
| B | 88 |
| C | 173 |
| D | 208 |
| Unknown | 19 |
| CEA | |
| < 5 | 89 |
| ≥ 5 | 367 |
| Missing value | 41 |
| Alkaline phosphatase | |
| < 133 | 287 |
| ≥ 133 | 208 |
| Missing value | 2 |
| Albumin | |
| < 35 | 144 |
| ≥ 35 | 351 |
| Missing value | 2 |
| 5-Fluorouracil | |
| PVI | 353 |
| Bolus | 144 |
| Number of metastatic sites | |
| 0 | 42 |
| 1 | 219 |
| 2 | 155 |
| 3 | 61 |
| > 3 | 20 |
| Metastatic site | |
| Liver | 361/497 |
| Lung | 134/497 |
| Peritoneal | 91/497 |
| Locoregional | 133/497 |
| Distant nodes | 118/497 |

for predicting response to chemotherapy (hazard ratio (HR) 2.27). Also, having performance status of 0 or 1, one metastasis or locally-advanced disease and administration of 5FU by PVI predicted for response in decreasing order. On analysis, albumin ≥ 35 (HR 1.63) approached statistical significance as a positive predictor for response. CEA ≥ 5 (HR 0.62) and presence of peritoneal metastases (HR 0.56) indicated a significantly decreased chance of a response to treatment.

### *Response rate according to site*

The most significant predictor for response in liver metastases was administration of 5FU by infusion (HR 3.01). Albumin ≥ 35 on presentation was as significant as performance status of 0 or 1 for predicting response in liver metastases, while the presence of peritoneal metastases made response unlikely (HR 0.34) (Table 4).

There was no positive predictor for response in peritoneal metastases on multivariate analysis. For response in lung metastases, the synchronous presence of peritoneal metastases approached statistical significance ($P$ = 0.07) for a decreased chance of response. Response was four times less likely in locoregional disease or distal nodes if CEA was raised on presentation. Response in locoregional disease was more likely if there was only one site of metastatic disease and if metastatic disease was present in distal nodes.

### *Survival*

Poor performance status, bolus administration of 5FU, CEA ≥ 5, albumin < 35, poor differentiation of initial tumour, presence of peritoneal metastases, alkaline phosphatase ≥ 133 and presence of distal nodal metastases were associated with a significant increased risk of mortality, in decreasing order (Table 5). The number of sites of metastases on presentation had no influence on overall survival, along with age, sex, site of primary and initial Dukes stage.

### DISCUSSION

In order to accurately assess the efficacy of alternative treatment regimens and compare different clinical trials, clarification of the clinical parameters which can predict response to chemotherapy and survival is required. The data for this review was collected from one centre and, although it involved different clinical trials and, therefore, different chemotherapy regimens, all regimens involved 5FU and the trials required similar entry criteria. The majority of the patients ($n$ = 302) were involved in trials assessing the effect of interferon, which has subsequently been shown to be of no benefit (Hill et al, 1995*a*, 1995*b*; Kosmidis et al, 1996; Recchia et al, 1996). Of the 497 patients, 99 received additional mitomycin C which, in one study, conferred benefit on response rate and failure-free survival, although no improvement in overall survival (Ross et al, 1997). Response rate for the whole group was similar to that previously reported (Kouri et al, 1992; Buroker et al, 1994; Conti et al, 1995).

The most important positive predictor for overall response to chemotherapy was the presence of liver metastases. Good performance status, only one site of metastasis and delivery of 5FU by PVI were also significant factors for response to chemotherapy. A raised CEA and presence of peritoneal metastases significantly decreased the chance of response.

By individual site, the opportunity for response was improved in the liver with infusional 5FU, good performance status and normal albumin. The synchronous presence of peritoneal metastases significantly decreased the chance of response in the liver or lung. A raised initial CEA was a negative predictive parameter for locoregional and distant nodal disease and there were no reliable predictive indicators for peritoneal metastases. Response to chemotherapy, as assessed by CT, was achieved in 40% of patients with liver metastases, 20.8% in lung metastases and only 10.4% patients with peritoneal disease. It has recently been found that there is a significantly higher level of thymidylate synthase (TS) mRNA expression and TS protein in lung metastases compared with liver metastases in colon carcinoma (Gorlick et al, 1998). As high TS expression is known to be predictive of a poor response to

© Cancer Research Campaign 1999

**Table 2** Response according to site of metastasis

| | Complete response *n* (%) | Partial response *n* (%) | Stable disease *n* (%) | Progressive disease *n* (%) | Non-evaluable* *n* (%) |
|---|---|---|---|---|---|
| Liver (*n* = 361) | 13 (3.8) | 124 (36.2) | 169 (49.3) | 37 (10.8) | 18 (5.0) |
| Lung (*n* = 134) | 3 (2.4) | 23 (18.4) | 85 (68.0) | 14 (11.2) | 9 (6.7) |
| Locoregional (*n* = 133) | 1 (0.9) | 16 (14.4) | 83 (74.8) | 11 (9.9) | 22 (16.5) |
| Nodal (*n* = 118) | 4 (3.7) | 16 (15.0) | 86 (80.4) | 1 (0.9) | 11 (9.3) |
| Peritoneal (*n* = 91) | – | 7 (10.4) | 46 (68.7) | 14 (20.9) | 24 (26.4) |

*Non-evaluable patients not included in the denominator for response by site

5FU (Johnston et al, 1995; Leichman et al, 1997; Yeh et al, 1998), it may explain the lack of response observed in the lung metastases compared with liver metastases in this cohort. It is probable that further mechanisms of resistance to chemotherapy regimens will be discovered in the future.

Poor performance status, treatment with bolus 5FU, raised CEA, decreased albumin, poor differentiation of tumour and raised alkaline phosphatase were all negative prognostic factors for overall survival. In addition, overall survival was poorest for those with peritoneal or distal nodal metastases, although the presence of more than one site of metastasis on presentation was not a significant predictor for survival.

A recent meta-analysis demonstrated that infusional 5FU was superior to bolus 5FU for achieving a response in advanced colorectal cancer and also conferred a slight increase in survival (Meta-analysis Group In Cancer, 1998). Raised CEA (Kouri et al, 1993), poor performance status (Kouri et al, 1992, 1993; Kosmidis et al, 1996) and poor differentiation of primary (Finan et al, 1985) have been reported previously as significant poor indicators for survival, as seen with our results. Contrary to our findings, age > 60 years (Kosmidis et al, 1996) and initial Dukes stage of primary tumour (Finan et al, 1985) have been found previously to be negative prognostic factors.

This study is unique for closely examining response and overall survival according to site of metastatic disease. However, certain weaknesses exist; slightly different 5FU-based chemotherapy regimens were used and it only provides predictive information of 5FU-treated patients. Its relevance to newer chemotherapy agents, such as irinotecan, which targets topoisomerase I instead of TS, will need to be studied in future trials. No account of the degree of replacement of the liver and lung by metastases was made and our present imaging techniques, including CT scanning, are limited in defining small foci of disease, such as in the peritoneum. In this series, 91 patients had evidence of peritoneal metastases. Of these, 26.4% had non-evaluable disease, 69% had stable disease, 21% had progressive disease and only 10% demonstrated a response to treatment, thus reflecting the difficulty of assessing peritoneal disease with CT scanning.

Nevertheless, metastatic involvement of the peritoneum, along with poor performance status, raised CEA, decreased albumin and treatment with bolus 5FU are important predictive indicators of poor response rate and increased mortality for patients with advanced colorectal cancer treated with 5FU. Response to chemotherapy is more likely if there are metastases in the liver and there is only one site of metastatic disease. Patients in clinical trials should be stratified according to clinical parameters, such as raised CEA, decreased albumin and site of metastasis on presentation, in addition to performance status, so that accurate comparisons between different treatment regimens can be made, and to ensure that poor-risk patients are not inappropriately offered potentially toxic chemotherapy.

**Table 3** Univariate analysis of prognostic factors for overall response

| | Number evaluable | Response rate (%) | *P* |
|---|---|---|---|
| Age | | | |
| <61 | 245 | 34.3 | |
| ≥61 | 244 | 34.4 | 0.974 |
| Sex | | | |
| Male | 309 | 35.3 | 0.575 |
| Female | 180 | 32.8 | |
| Performance status | | | |
| 0–1 | 351 | 39.3 | 0.0002 |
| 2–4 | 131 | 21.4 | |
| Site of primary | | | |
| Colon | 320 | 35.3 | 0.540 |
| Rectum | 169 | 32.5 | |
| Differentiation | | | |
| Well/Mod | 401 | 34.7 | |
| Poor | 64 | 35.9 | 0.843 |
| CEA | | | |
| < 5 | 85 | 42.4 | 0.085 |
| ≥ 5 | 363 | 32.5 | |
| Alkaline phosphatase | | | |
| < 133 | 280 | 38.2 | 0.034 |
| ≥ 133 | 207 | 29.0 | |
| Albumin | | | |
| < 35 | 144 | 20.1 | |
| ≥ 35 | 343 | 40.2 | < 0.0001 |
| 5-Fluorouracil | | | |
| PVI | 345 | 38.6 | 0.003 |
| Bolus | 144 | 24.3 | |
| Number of metastatic sites | | | |
| 0–1 | 255 | 42.4 | < 0.0001 |
| ≥ 2 | 234 | 25.6 | |
| Liver metastases | | | |
| No | 133 | 28.6 | |
| Yes | 356 | 36.5 | 0.100 |
| Lung metastases | | | |
| No | 357 | 37.5 | 0.015 |
| Yes | 132 | 25.8 | |
| Peritoneal metastases | | | |
| No | 398 | 37.2 | 0.006 |
| Yes | 91 | 22.0 | |
| Nodal metastases | | | |
| No | 368 | 36.7 | 0.059 |
| Yes | 121 | 27.3 | |

© Cancer Research Campaign 1999

**Table 4** Multivariate analysis of response rate – overall and according to site of metastasis

| Test | Factor | *n* | Hazard ratio | 95% CI |
|---|---|---|---|---|
| Overall response (*n* = 420) | PS | | | |
| | 0–1 | 307 | 1.91 | 1.1–3.3 |
| | 2–4 | 113 | | |
| | CEA | | | |
| | < 5 | 80 | | |
| | ≥ 5 | 340 | 0.62 | 0.35–1.1 |
| | Albumin | | | |
| | < 35 | 122 | | |
| | ≥ 35 | 298 | 1.63 | 0.95–2.81 |
| | 5 FU | | | |
| | PVI | 319 | 1.84 | 1.1–3.1 |
| | Bolus | 101 | | |
| | Metastatic sites | | | |
| | 0–1 | 216 | 1.85 | 1.1–3.0 |
| | > 1 | 204 | | |
| | Liver metastases | | | |
| | No | 114 | | |
| | Yes | 306 | 2.27 | 1.33–3.89 |
| | Peritoneal metastases | | | |
| | No | 338 | | |
| | Yes | 82 | 0.56 | 0.29–1.08 |
| Liver response (*n* = 299) | PS | | | |
| | 0–1 | 210 | 1.91 | 1.03–3.53 |
| | 2–4 | 89 | | |
| | Albumin | | | |
| | < 35 | 97 | | |
| | ≥ 35 | 202 | 1.91 | 1.03–3.53 |
| | 5 FU | | | |
| | PVI | 219 | 3.01 | 1.62–5.6 |
| | Bolus | 80 | | |
| | Peritoneal metastases | | | |
| | No | 239 | | |
| | Yes | 60 | 0.34 | 0.17–0.67 |
| Lung response (*n* = 109) | Peritoneal metastases | | | |
| | No | 87 | | |
| | Yes | 22 | 0.27 | 0.05–1.37 |
| Local response (*n* = 150) | CEA | | | |
| | < 5 | 33 | | |
| | ≥ 5 | 117 | 0.25 | 0.09–0.68 |
| | Metastatic sites | | | |
| | 0–1 | 86 | 3.22 | 0.97–11.1 |
| | > 1 | 64 | | |
| | Nodal metastases | | | |
| | No | 113 | | |
| | Yes | 37 | 3.13 | 0.96–10.2 |
| Nodal response (*n* = 101) | CEA | | | |
| | < 5 | 15 | | |
| | ≥ 5 | 86 | 0.25 | 0.07–0.86 |

**Table 5** Overall survival – hazard ratios for mortality

| Factor | Hazard ratio | 95% CI |
|---|---|---|
| Performance Status | | |
| 0–1 | | |
| 2–4 | 2.10 | 1.60–2.70 |
| Differentiation | | |
| Well/Mod | | |
| Poor | 1.51 | 1.10–2.10 |
| CEA | | |
| < 5 | | |
| ≥ 5 | 1.72 | 1.30–2.30 |
| 5FU | | |
| PVI | | |
| Bolus | 1.90 | 1.50–2.40 |
| Alkaline phosphatase | | |
| < 133 | | |
| ≥ 133 | 1.40 | 1.10–1.80 |
| Albumin | | |
| < 35 | 1.69 | 1.32–2.17 |
| ≥ 35 | | |
| Peritoneal metastases | | |
| No | | |
| Yes | 1.46 | 1.10–1.90 |
| Nodal metastases | | |
| No | | |
| Yes | 1.35 | 1.06–1.73 |

### REFERENCES

Advanced Colorectal Cancer Meta-Analysis Project (1992) Modulation of Fluorouracil by leucovorin in patients with advanced colorectal cancer: Evidence in terms of response rate. *J Clin Oncol* **10**: 896–903

Allen-Mersh TG, Earlam S, Fordy C, Abrams K and Houghton J (1994) Quality of life and survival with continuous hepatic-artery floxuridine infusion for colorectal liver metastases. *Lancet* **344**: 1255–1260

Buroker T, O'Connnell M, Wieand S, Krook JE, Gerstner JB, Mailliard JA, Schaefer PL, Levitt R, Kardinal CG and Gesme DH (1994) Randomized comparison of two schedules of fluorouracil and leucovorin in the treatment of advanced colorectal cancer. *J Clin Oncol* **12**: 14–20

Cancer Research Campaign (1998) Factsheet 1.3 (incidence of cancer in males in UK) and 1.4 (incidence of cancer in females in UK).

Chang AE, Steinberg SM, Culnane M and White DE (1989) Determinants of survival in patients with unresectable colorectal liver metastases. *J Surg Oncol* **40**: 245–251

Conti JA, Kemeny NE, Saltz LB, Andre AM, Grossano DD and Bertino JR (1995) Continuous infusion fluorouracil/leucovorin and bolus mitomycin C as a salvage regimen for patients with advanced colorectal cancer. *Cancer* **75**: 769–774

Finan PJ, Marshall RJ, Cooper EH and Giles GR (1985) Factors affecting survival in patients presenting with synchronous hepatic metastases from colorectal cancer: a clinical and computer analysis. *Br J Surg* **72**: 373–377

Fountzilas G, Gossios K, Zisiadis A, Svarna E, Skarlos D and Pavlidis N (1996). Prognostic variables in patients with advanced colorectal cancer treated with fluorouracil and leucovorin-based chemotherapy. *Med & Ped Oncol* **26**: 305–317

Gorlick R, Metzger R, Danenberg KD, Salonga D, Miles JS, Longo GSA, Fu J, Banerjee D, Klimstra D, Jhanwar S, Danenberg PV, Kemeny N and Bertino JR (1998) Higher levels of thymidylate synthase gene expression are observed in pulmonary as compared with hepatic metastases of colorectal adenocarcinoma. *J Clin Oncol* **16**: 1465–1469

Hill M, Norman A, Cunningham D, Findlay M, Watson M, Nicolson V, Webb A, Middleton G, Ahmed F, Hickish T, Nicolson M, O'Brien M, Iveson T, Iveson A and Evans C (1995*a*) Impact of protracted venous infusion fluorouracil with or without interferon Alfa-2b on tumor response, survival and quality of life in advanced colorectal cancer. *J Clin Oncol* **13**: 2317–2323

Hill M, Norman A, Cunningham D, Findlay M, Nicolson V, Hill A, Iveson A, Evans C, Joffe J, Nicolson M and Hickish T (1995*b*) Royal Marsden phase III trial of Fluorouracil with or without interferon Alfa-2b in advanced colorectal cancer. *J Clin Oncol* **13**: 1297–1302

Johnston PG, Lenz HJ, Leichman CG, Danenberg KD, Allegra CJ, Danenberg PV and Leichman L (1995) Thymidylate synthase gene and protein expression correlate and are associated with response to 5-fluorouracil in human colorectal and gastric tumors. *Cancer Res* **55**: 1407–1412

Kosmidis PA, Tsavaris N, Skarlos D, Theocharis D, Samantas E, Pavlidis N, Briassoulis E and Fountzilas G (1996) Fluorouracil and leucovorin with or without interferon Alfa-2b in advanced colorectal cancer: analysis of a prospective randomized phase III trial. *J Clin Oncol* **14**: 2682–2687

Kouri M, Pyrhonen S and Kuusela P (1992) Elevated CA19-9 as the most significant prognostic factor in advanced colorectal carcinoma. *J Surg Oncol* **49**: 78–85

 *© Cancer Research Campaign 1999*

Kouri M, Nordling S, Kuusela P and Pythonen S (1993) Poor prognosis associated with elevated serum CA 19-9 level in advanced colorectal carcinoma, independent of DNA ploidy or SPF. *Eur J Cancer* **29A**: 1691–1696

Leichman CG, Lenz HJ, Leichman L, Danenberg K, Baranda J, Groshen S, Bowsell W, Metzger R, Tan M and Danenberg PV (1997) Quantitation of intratumoral thymidylate synthase expression predicts for disseminated colorectal cancer response and resistance to protracted-infusion fluorouracil and weekly leucovorin. *J Clin Oncol* **15**: 3223–3229

Mayer RJ (1992) Chemotherapy for metastatic colorectal cancer. *Cancer* **70**: 1414–1424

Meta-analysis Group In Cancer (1998) Efficacy of intravenous continuous infusion of fluorouracil compared with bolus administration in advanced colorectal cancer. *J Clin Oncol* **16**: 301–308

Miller AB, Hoogstraaten B, Staquet M and Winkler A (1981) Reporting results of cancer treatment. *Cancer* **47**: 207–214

Nordic Gastrointestinal Tumour Adjuvant Therapy Group (1992) Expectancy or primary chemotherapy in patients with advanced asymptomatic colorectal cancer: a randomized trial. *J Clin Oncol* **10**: 904–911

Poon MA, O'Connell MJ, Moertel CG, Wieand HS, Cullinan SA, Everson LK, Krook JE, Mailliard JA, Laurie J, Tschetter LK and Wiesenfeld M (1989) Biochemical modulation of Fluorouracil: evidence of significant improvement of survival and quality of life in patients with advanced colorectal carcinoma. *J Clin Oncol* **7**: 1407–1418

Recchia F, Nuzzo A, Lalli A, Lombardo M, Di-Lullo L, Fabiani F, Fanini R, Venturoni L, Torchio P and Peretti G (1996) Randomized trial of 5-Fluorouracil and high-dose folinic acid with or without alpha-2b interferon in advanced colorectal cancer. *Am J Clin Oncol* **19**: 301–304

Ross P, Norman A, Cunningham D, Webb A, Iveson T, Padhani A, Prendiville J, Watson M, Massey A and Oates J (1997) A prospective randomised trial of protracted venous infusion (PVI) 5-FU with or without mitomycin C (MMC) in advanced colorectal cancer. *Annals Oncol* **8**: 995–1001

Scheithauer W, Rosen H, Kornek GV, Sebesta C and Depisch D (1993) Randomised comparison of combination chemotherapy plus supportive care with supportive care alone in patients with metastatic colorectal cancer. *BMJ* **306**: 752–755

Seymour MT, Slevin ML, Kerr DJ, Cunningham D, James RD, Ledermann JA, Perren TJ, McAdam WA, Harper PG, Neoptolemos JP, Nicholson M, Duffy AM, Stephens RJ, Stenning SP and Taylor I (1996) Randomized trial assessing the addition of interferon alpha-2a to fluorouracil and leucovorin in advanced colorectal cancer. *J Clin Oncol* **14**: 2280–2288

Stangl R, Altendorf-Hofmann A, Charnley R and Scheele J (1994) Factors influencing the natural history of colorectal liver metastases. *Lancet* **343**: 1405–1410

Steinberg J, Erlichman C, Gadalla T, Fine S and Wong A (1992) Prognostic factors in patients with metastatic colorectal cancer receiving 5-fluorouracil and folinic acid. *Eur J Cancer* **28**A(ii): 1817–1820

Webb A, Scott-Mackie P, Cunningham D, Norman A, Andreyev J, O'Brien M and Bensted J (1995) The prognostic value of CEA, bHCG, AFP, CA125, CA19-9 and C-erb B-2, bHCG immunohistochemistry in advanced colorectal cancer. *Annals Oncol* **6**: 581–587

Yeh KH, Shun CT, Chen CL, Lin JT, Lee WJ, Lee PH, Chen YC and Cheng AL (1998) High expression of thymidylate synthase is associated with the drug resistance of gastric carcinoma to high dose 5-fluorouracil-based systemic chemotherapy. *Cancer* **82**: 1626–1631

*© Cancer Research Campaign 1999*
